# A Giant Solid-Cystic Gastric Inflammatory Myofibroblastic Tumor: A Case Report and Literature Review

**DOI:** 10.7759/cureus.37167

**Published:** 2023-04-05

**Authors:** Gunjan Desai, Deepak M Parikh, Prasad K Wagle

**Affiliations:** 1 Gastrointestinal Surgery, Asian Cancer Institute and ACI Cumballa Hill Hospital, Mumbai, IND; 2 Gastrointestinal Surgery, Lilavati Hospital and Research Centre, Mumbai, IND; 3 Head and Neck Surgery, Asian Cancer Institute and ACI Cumballa Hill Hospital, Mumbai, IND; 4 Head and Neck Surgery, Lilavati Hospital and Research Centre, Mumbai, IND

**Keywords:** gastric submucosal lesions, gastrointestinal stromal tumor, anaplastic lymphoma kinase, inflammatory myofibroblastic tumor, inflammatory pseudotumor

## Abstract

An inflammatory myofibroblastic tumor (IMT) is a very rare tumor of mesenchymal origin with unclear etiopathogenesis, no unique diagnostic features, and no specific management protocol. It is often confused with inflammatory pseudotumor in literature, and the distinction needs further study. The average size, recurrence risk, and metastatic potential differ as per the site of origin. The abdomen is a very rare site for IMTs. Hepatic IMTs (H-IMTs) are reported to be solid tumors with sizes ranging from 1 cm to 20 cm in literature, and gastric IMTs (G-IMTs) range from 3 cm to 10 cm in size and can be solid-cystic. We report here a case of a 36-year-old gentleman with a 34x27x17 cm solid-cystic lesion in the lesser sac with loss of fat planes with stomach and left hemi-liver. The patient was managed by complete surgical resection of the lesion with wedge gastrectomy and wedge hepatectomy and recovered uneventfully. To our knowledge and based on our literature review, this case presents the largest reported and solid-cystic G-IMT with the involvement of left hemi-liver in a young gentleman and discusses its management as well as the relevant literature on this rare entity. This clinical presentation of G-IMT should be kept in the differential diagnosis in a relevant case presenting in the future. Immunohistochemistry is a must to establish the diagnosis, and surgical resection to negative margins is the management option of choice in resectable cases.

## Introduction

An inflammatory myofibroblastic tumor (IMT) is a very rare tumor with unclear etiopathogenesis and no specific management protocol [1,2]. Also, there is a lack of clarity on the distinction between inflammatory pseudotumor (IPT) and IMT across the literature [2,3]. First identified in lungs and more common in pediatric and adolescent age groups, there are case reports of this tumor occurring in various organs such as salivary glands, skin, spleen, orbit, prostate, breast, uterus, and urinary bladder with very rare occurrence in the large bowel, stomach, and liver as well as extra-hepatic bile duct [3-5].

The average size, recurrence risk, and metastatic potential differ as per the site of origin. Hepatic IMTs (H-IMTs) are reported to be solid tumors with sizes ranging from 1 cm to 20 cm across the case reports and series in the literature [3,4,6]. G-IMTs range from 3 cm to 10 cm in size and can be solid-cystic [5-7]. To our knowledge and based on our literature review, this case presents the largest reported gastric IMT (G-IMT) with the involvement of left hemi-liver in a young gentleman and discusses its management as well as the relevant literature on this rare entity.

## Case presentation

**Figure 1:**
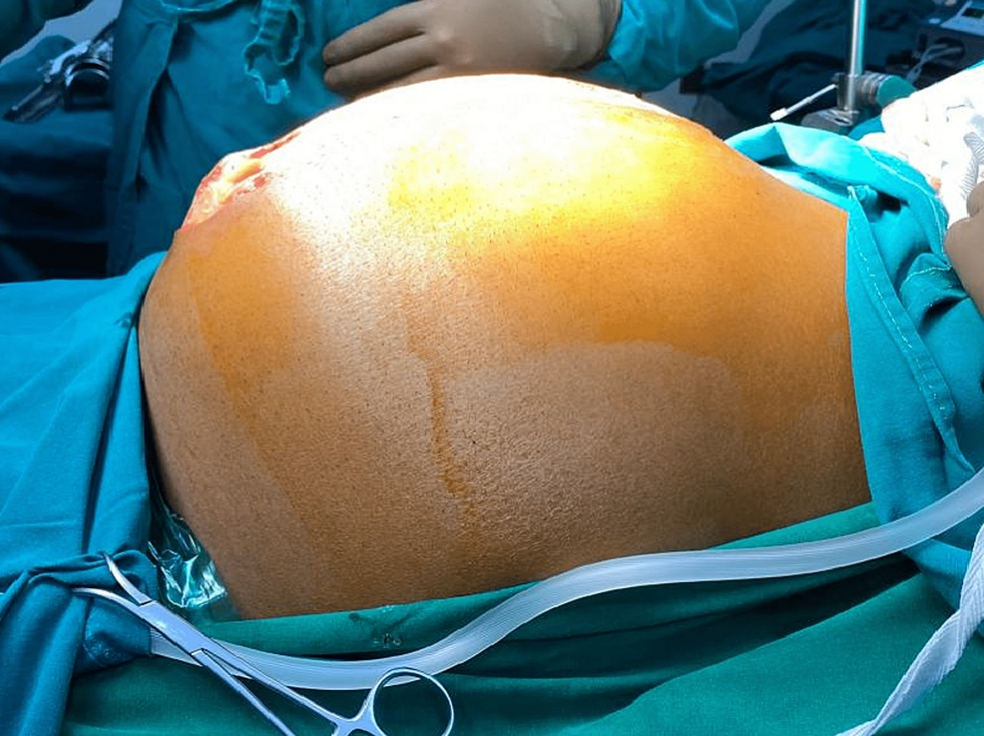
The patient's abdomen is massively distended due to the tumor

A 36-year-old gentleman presented to the emergency department of our tertiary care center with breathlessness, severe abdominal distension, and abdominal pain. He also had early satiety, weight loss, and low-grade fever for the last two months and noticed his gradually increasing abdominal distension in one to two months as shown in Figure 1.

He had no previous history of abdominal pain. There was no history of alcohol or tobacco consumption, no co-morbidities, and no significant family history. There was no history of abdominal trauma or surgery in the past. On clinical examination, he had pallor, persistent tachycardia, and a respiratory rate of 30-40 breaths/minute. He had an ultrasound of his abdomen from another hospital that had reported a solid-cystic lesion in the abdomen and an ultrasound-guided biopsy that had reported the lesion as low-grade myxoid sarcoma - Fédération Nationale des Centres de Lutte Contre Le Cancer (FNCLCC) grade 1 [8].

**Figure 2:**
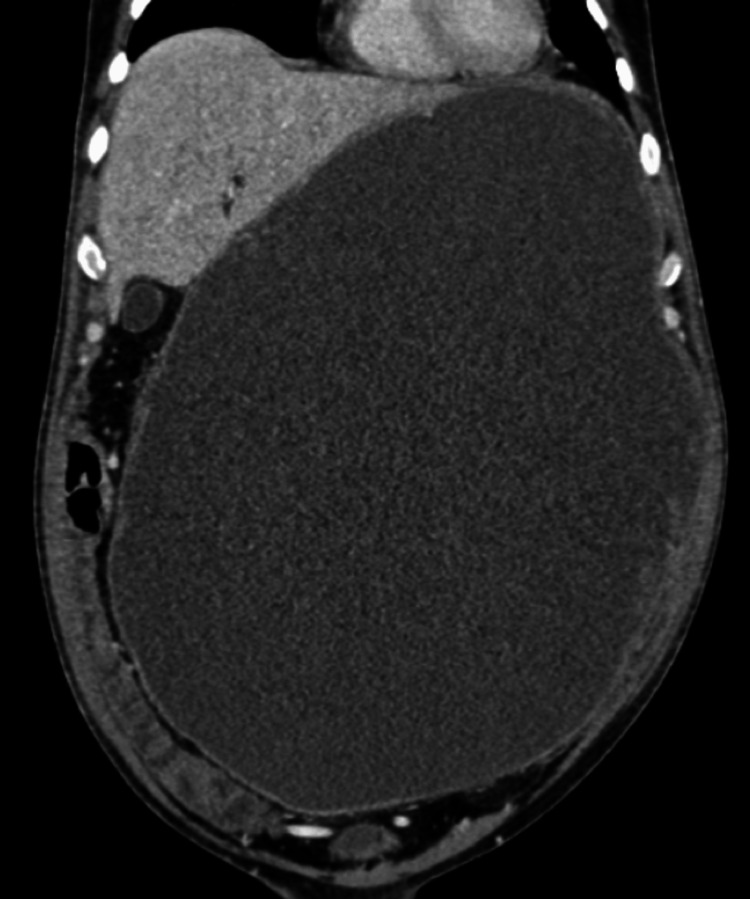
CECT coronal image showing the large cystic lesion with complete loss of fat planes to the left lateral liver sector CECT, contrast-enhanced computerized tomography

**Figure 3:**
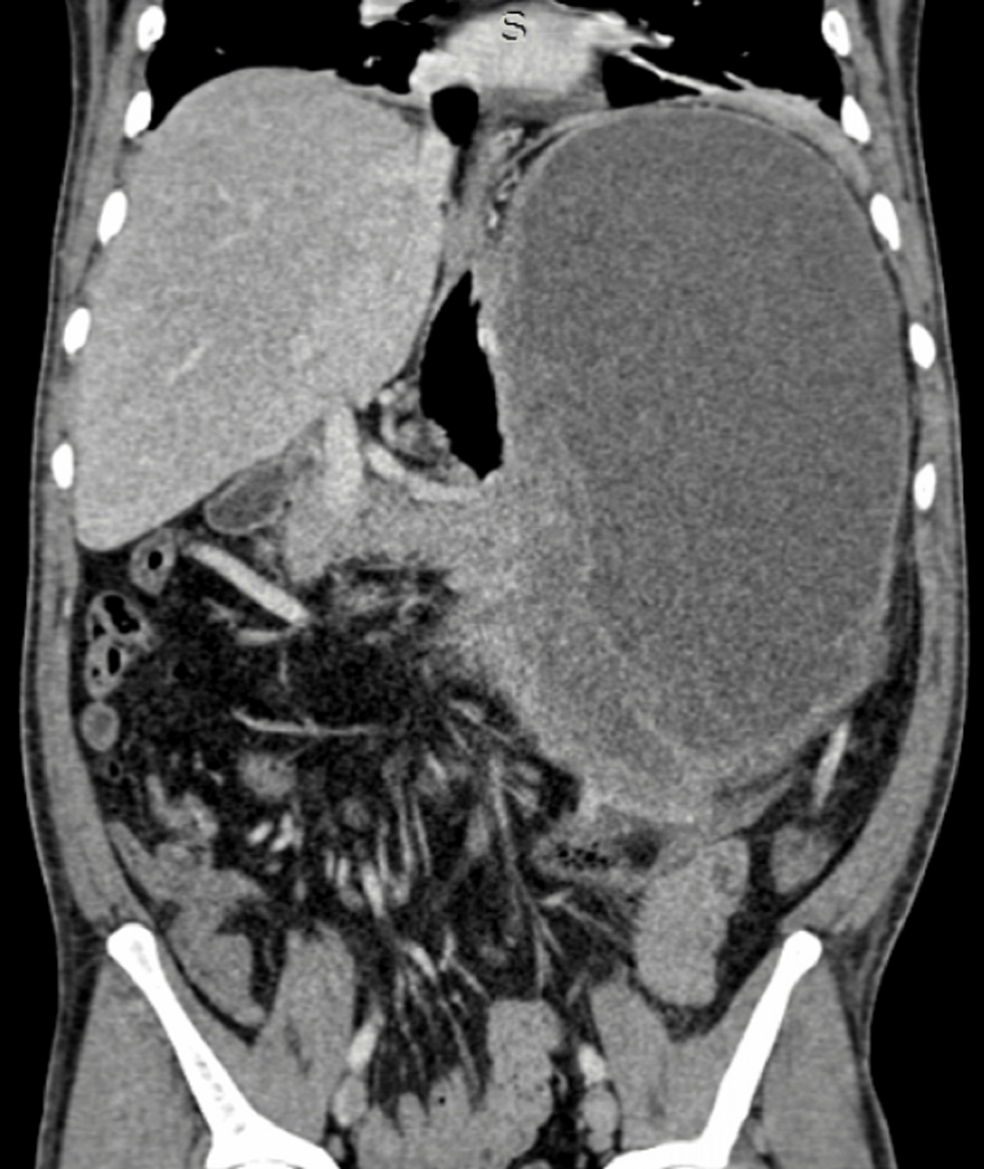
CECT coronal image showing the large solid-cystic lesion in relation to the stomach and the pancreas CECT, contrast-enhanced computerized tomography

After resuscitation and blood investigations, a contrast-enhanced computerized tomography (CECT abdomen with IV contrast) of the abdomen was performed which showed a 34x27x17 cm solid-cystic lesion in the lesser sac with loss of fat planes between the lesion and the stomach as well as left hemi-liver as shown in Figures 2 and 3.

**Figure 4:**
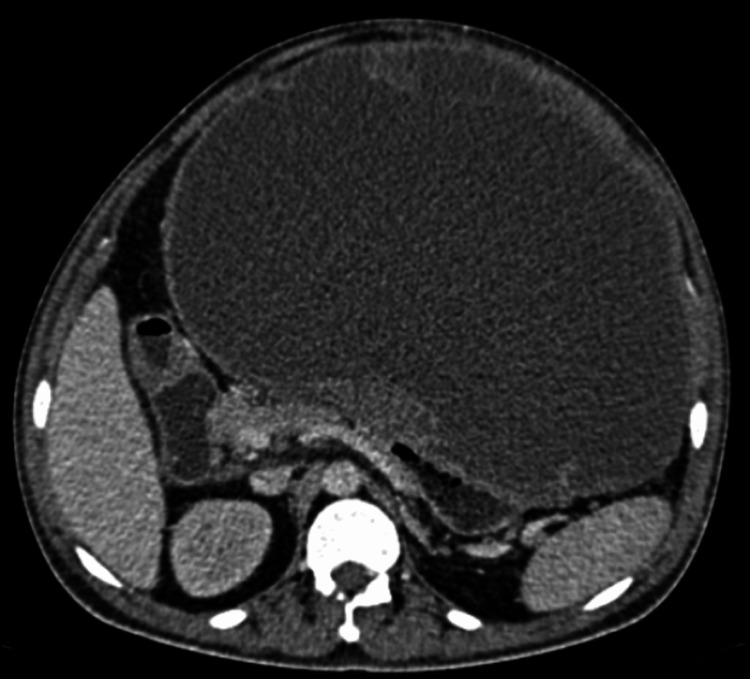
CECT axial image showing the large solid-cystic lesion in relation to the duodenum, pancreas, and the spleen CECT, contrast-enhanced computerized tomography

Duodenum and colon were closely abutted by the lesion and the pancreas looked compressed as shown in Figure 4.

**Figure 5:**
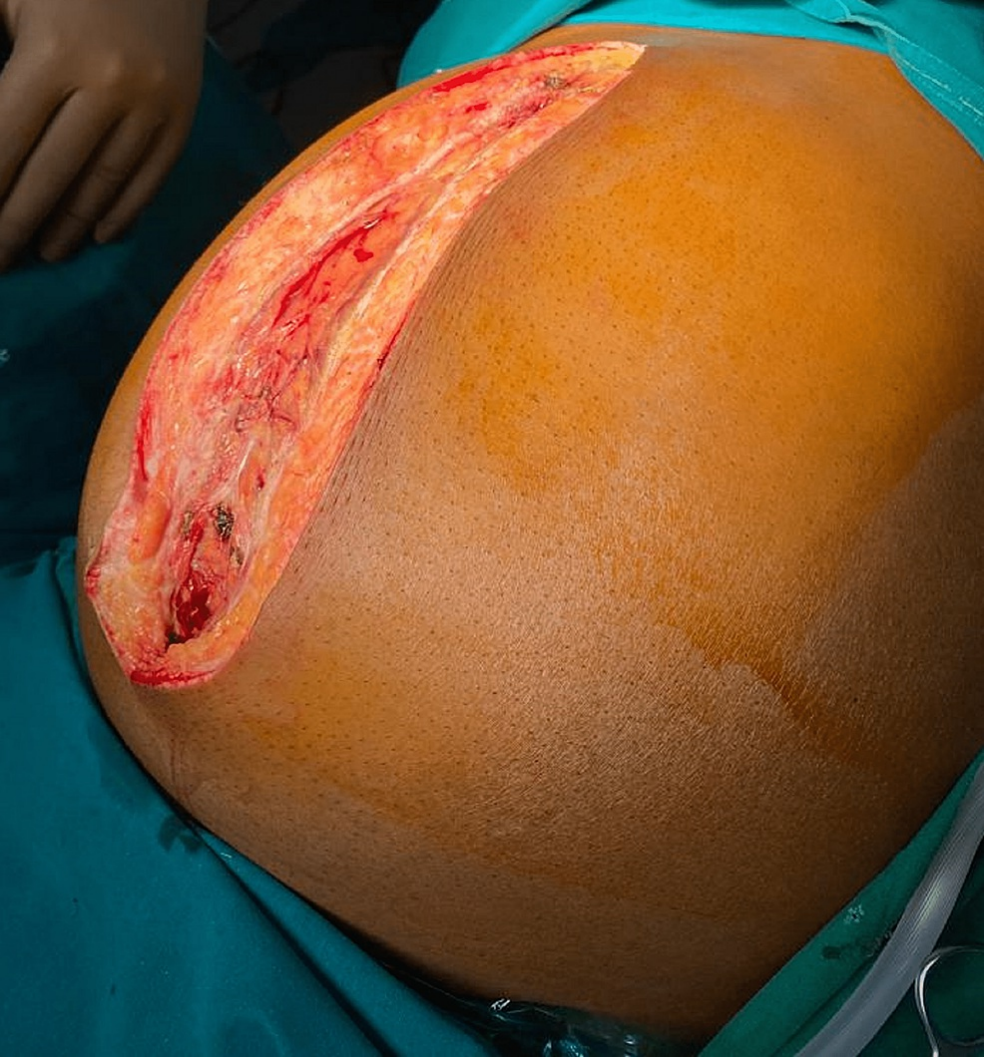
Vertical midline incision to approach the lesion

**Figure 6:**
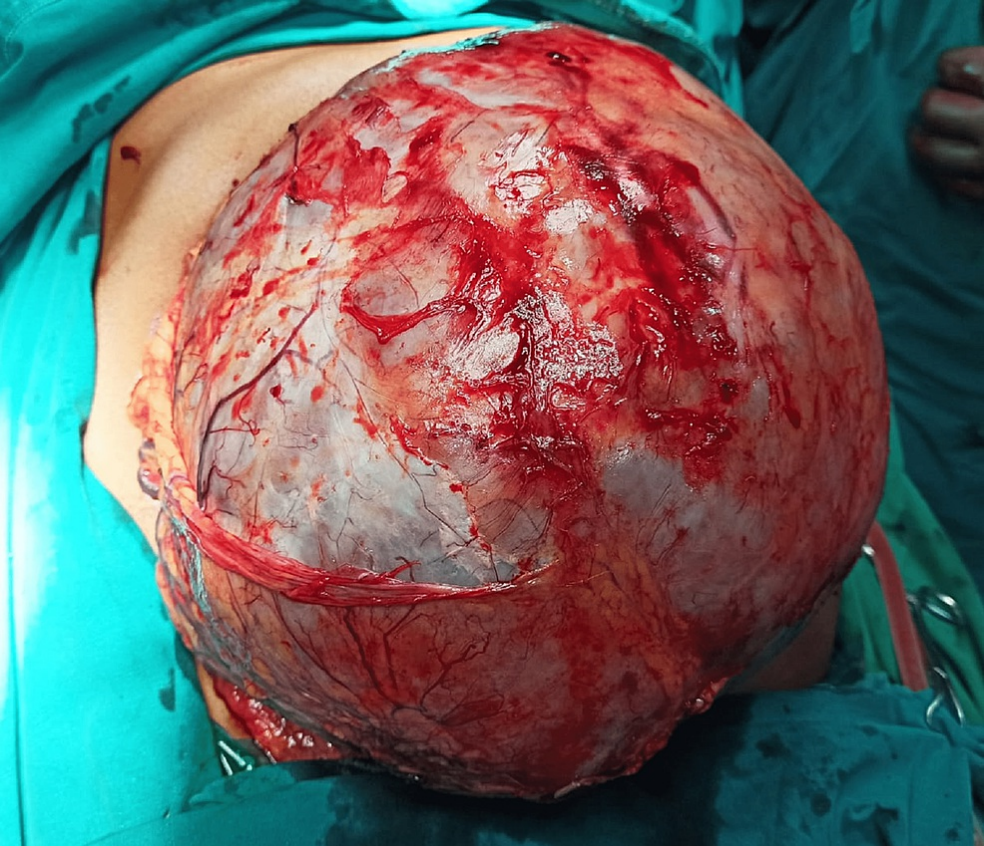
The intra-operative appearance of the dissected tumor

His hemoglobin was 8 gm/dl, and he had leukocytosis with neutrophilia as well as elevated C-reactive protein levels. Other parameters were within normal limits. After ascertaining anesthesia fitness, we proceeded with a midline abdominal laparotomy as seen in Figures 5 and 6.

Intra-operatively, the lesion was resected along with a wedge of the stomach and a wedge of liver en-masse with the tumor. Post-operatively he recovered well and was discharged on the eighth post-operative day uneventfully.

**Figure 7:**
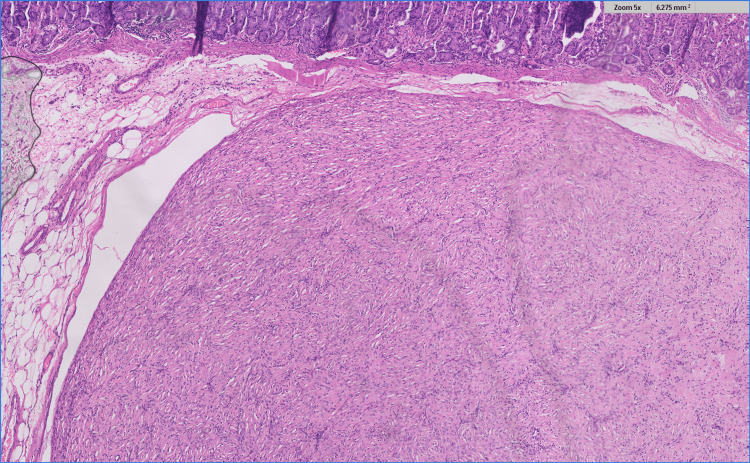
The histopathological image with hematoxylin and eosin staining shows a gastric sub-mucosal circumscribed cellular spindle cell tumor with occasional mononuclear inflammatory infiltrate

On histopathology as seen in Figure 7, there was a solid-cystic lesion in the sub-mucosal layer of the stomach with intact mucosa, with a varying wall thickness of 0.3-1 cm and areas of solid component measuring 5.5x3x3 cm extending into the liver parenchyma. The liver margin was free from the tumor, and the gastric mucosa was free from the tumor. The tumor was pseudo-capsulated, with spindle-shaped myofibroblastic tumor cells in a compact fascicular arrangement. The cells had spindle-shaped nuclei with eosinophilic cytoplasm. Minimal lymphoid follicle formations and plasma cell groups were noted in some sections more intense in the tumor periphery.

**Figure 8:**
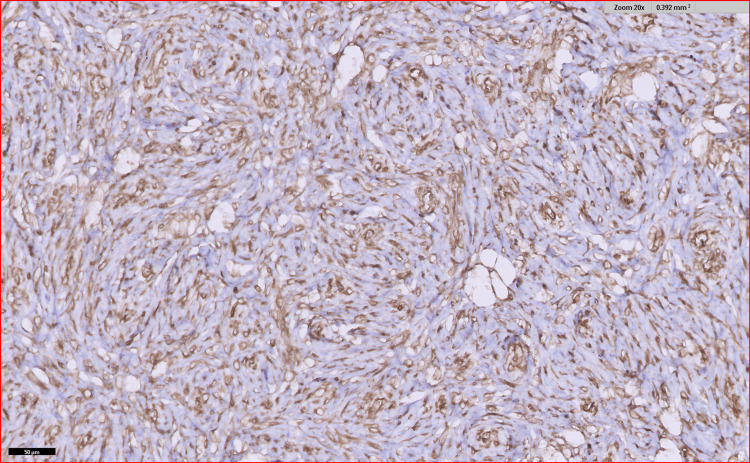
IHC for vimentin showing focal positivity IHC, immunohistochemistry

**Figure 9:**
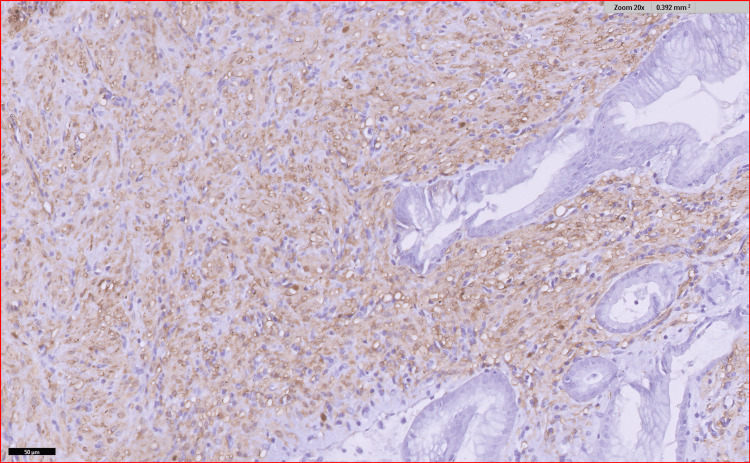
IHC for SMA showing positivity IHC, immunohistochemistry; SMA, smooth muscle actin

**Figure 10:**
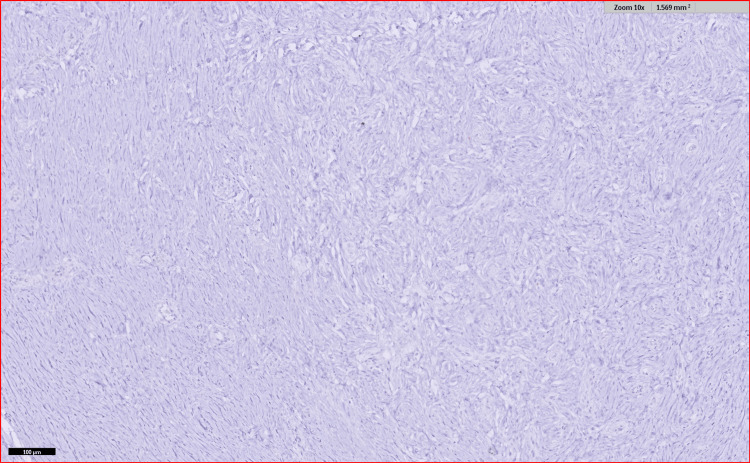
IHC for ALK was negative IHC, immunohistochemistry; ALK, anaplastic lymphoma kinase

**Figure 11:**
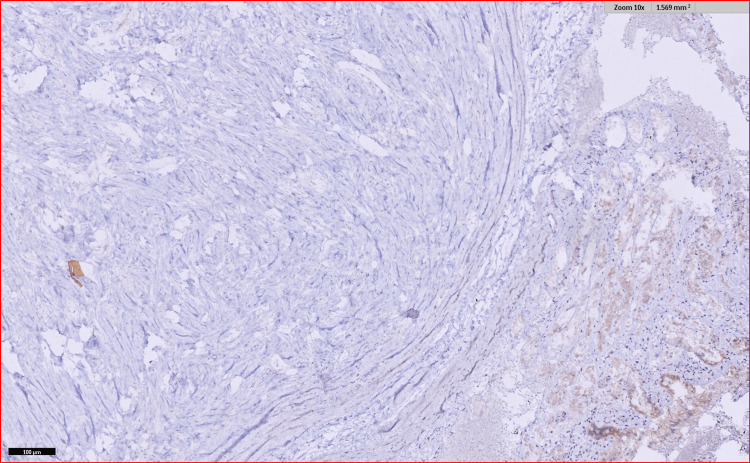
IHC discovered on DOG1 was negative IHC, immunohistochemisty; DOG1, discovered on gastrointestinal stromal tumor 1

Immunohistochemistry (IHC) markers revealed the tumor to be negative for S-100, CD 117, anaplastic lymphoma kinase (ALK), discovered on gastrointestinal stromal tumor 1 (DOG1), human melanoma black 45 (HMB 45), and CD 34 whereas, it was positive for smooth muscle actin (SMA) as well as vimentin and desmin was focally positive as seen in Figures 8, 9, 10, and 11.

The ki-67 index was 9%. The final histopathological diagnosis was a low-grade G-IMT with complete resection (R0) status and the patient needed no further treatment. He is doing well at six-month follow-up. 

## Discussion

IMTs are very rare tumors with unknown actual incidence due to their rarity. They were thought to be synonymous with IPT, which has an estimated incidence of 0.7% in the literature [1,2]. However, IPTs include a lot of non-neoplastic fibro-inflammatory lesions, whereas IMTs have been found to have the ability to recur and metastasize and have distinct clinic-pathological and molecular features and hence are considered distinct tumors of intermediate biological potential [2,3].

These are tumors of mesenchymal origin, and etiopathogenesis is not clear. Possible etiological factors include infections such as Epstein-Barr virus, *Coxiella burnetii*, *Corynebacterium equi*, *Bacillus sphaericus*, and Mycobacterium avium intracellulare, trauma, past surgery, and association with inflammatory and autoimmune diseases, such as autoimmune pancreatitis, gout, and primary sclerosing cholangitis. The final common pathophysiologic mechanism behind all these etiological factors is an uncontrolled proliferation of myofibroblasts leading to tumor formation [3,4,9]. Recent studies have identified a gene rearrangement in the ALK locus on chromosome 2p23, and this has been identified in IHC in 50-60% of cases. ALK expression is considered protective and a good prognostic factor in these patients [9,10].

Clinically, there are no specific clinical manifestations and no gender predisposition for IMTs. However, G-IMTs and H-IMTs are found to be slightly more common in females. On the other hand, abdominal IPTs are more common in males as solitary lesions in the right hemi-liver in the third and fourth decades [1-3]. G-IMTs are usually seen in children and young adults and may present with abdominal pain or distension, abdominal lump, fever of unknown origin, hematemesis, or melena [6,7,10]. Loss of weight and appetite is rarely reported. There is only one report of G-IMT with peritoneal dissemination at presentation [11]. Another patient had an IMT at the gastric remnant after a prior gastrectomy for an unrelated diagnosis [12]. There is a case report of H-IMT in the fetal period leading to an oncological emergency in the pregnancy [13]. The lesion has been reported in the gastric sub-mucosa in all cases and commonly in the pre-pyloric region, gastric body, and gastric antrum compared to the proximal stomach [6,10-12].

On investigations, the patients may have anemia, thrombocytosis, leukocytosis with neutrophilia, elevated markers of inflammation such as erythrocyte sedimentation rate and C-reactive protein, and polygonal hyper-gammaglobulinemia. However, these features can be present in both IPTs and IMTs [2,3,7,8]. They lack specific imaging features that can be diagnostic. IMTs are usually solitary, hypo-echoic mass or rarely hyper-echoic mass on ultrasound and hypo-intense with variable contrast uptake on CECT abdomen and pelvis leading to no enhancement, segregated or marginal enhancement, and rarely complete tumor filling. They have a low signal on T1W magnetic resonance imaging and a medium-to-high signal on T2W images. None of these imaging features are diagnostic of IMTs [7,8,10,14].

Our patient had a predominantly cystic lesion with few solid components with involvement of the stomach and left hemi-liver leading to a probable diagnosis of G-IMT or H-IMT. However, owing to the classical gastric sub-mucosal location of the tumor with spared mucosa, the final pathological diagnosis was G-IMT. Histopathological evaluation of a biopsy or the surgical specimen with IHC is a must to establish the diagnosis of this rare tumor. Based on clinicopathological and imaging features without IHC, it can easily be confused with gastrointestinal stromal tumor (GIST), immunoglobulin subtype G4 (IgG4)-related disease as well as liposarcoma, follicular dendritic cell sarcoma, solitary or calcifying fibrous tumor, and metastatic or spindle cell sarcoma or sarcomatoid carcinoma [10,14,15].

Pathologically, IMTs can have one or more of the following three patterns: myxoid/vascular patterns with loosely arranged plump spindle cells in a myxoid stroma with prominent vascular pattern and less lymphoplasmacytic infiltrate, spindle cell pattern with fascicular or storiform architecture, and hypo-cellular fibrous pattern with elongated spindle cells and scattered lymphocytes in a collagenous stroma. These three patterns can also be present in a single patient.

The most common pathological pattern seen in IMTs is the myxoid/vascular pattern [7]. On IHC, the tumor is negative for DOG1 and CD 117; thus, differentiating it from GIST, lacks IgG4 expressing plasma cells or has less IgG4 plasma cell infiltration and no obliterative phlebitis; thereby, differentiating it from IgG4-related disease and can have both B and T cells that differentiate it from lymphomas that are clonal tumors with either B or T cells. It is negative for CD34 and S-100 as well. The IMTs usually stain positive for one or more of SMA, vimentin, desmin, and ALK in 50-60% of cases, and this entire IHC study is necessary to make a diagnosis as summarized in Table 1 [6,7,10,14,15].

**Table 1:** Immunohistochemistry markers used in the differential diagnosis of this rare entity DOG1, discovered on gastrointestinal stromal tumor 1; SMA, smooth muscle actin; ALK, anaplastic lymphoma kinase; GIST, gastrointestinal stromal tumor; IPT, inflammatory pseudotumor; IMT, inflammatory myofibroblastic tumor

Immunohistochemistry markers	Differential diagnosis
DOG1 and/or CD 117	GIST
IgG4 expression	IPT, IgG4-related disease
CD 34	Hematopoietic stem cell
S-100	Nerve sheath tumors and melanocytic tumors
SMA	Myoepithelial differentiation
Desmin	Skeletal muscle
Vimentin	Myoblasts and connective tissue
ALK	IMT

Owing to its rarity, there are no definite management protocols for G-IMT. However, across the literature, surgical resection to negative margins has been the treatment of choice [6,7]. Occasional reports of spontaneous regression, chemotherapy, high-dose steroid therapy, non-steroidal anti-inflammatory drug therapy, and radiation have been published, but these tumors seem to be IPTs and not IMTs. Local recurrence rates are up to 25%, and large tumor size at presentation, abdominopelvic location, and older age are the risk factors [2,6,7,12,15]. On the other hand, metastasis has been reported in less than 5% of cases, and younger age and lack of ALK expression are the risk factors [9,10]. Our patient has been doing well with no recurrence or metastatic disease at the six-month follow-up.

## Conclusions

To our knowledge, this is the first reported case in literature where a patient presented with a giant low-grade G-IMT of a size of 34 cm and a significant cystic component. This clinical presentation of G-IMT should be kept in the differential diagnosis in a relevant case presenting in the future. IHC is a must to establish the diagnosis, and surgical resection to negative margins is the management option of choice in resectable cases. 
